# Planar cell polarity, cilia, and human disease

**DOI:** 10.1186/2046-2530-1-S1-K2

**Published:** 2012-11-16

**Authors:** JB Wallingford

**Affiliations:** 1 University of Texas, Austin, USA

## 

The planar cell polarity (PCP) proteins govern diverse cellular behaviors, are essential for embryonic development, and are associated in a variety of human birth defects, including ciliopathies. While many PCP proteins have been extensively characterized, the “PCP effector” proteins, Inturned, Fuzzy and Fritz, remain largely unstudied. We have shown that each of these novel proteins is broadly essential for ciliogenesis in vertebrate animals. Here, we will discuss our efforts to combine bioinformatics, genomics, mouse genetics, and in vivo time-lapse imaging of intraflagellar transport to understand the mechanisms by which the PCP proteins govern cilia structure and function.

